# Predictors of Condom Use among Peer Social Networks of Men Who Have Sex with Men in Ghana, West Africa

**DOI:** 10.1371/journal.pone.0115504

**Published:** 2015-01-30

**Authors:** LaRon E. Nelson, Leo Wilton, Thomas Agyarko-Poku, Nanhua Zhang, Yuanshu Zou, Marilyn Aluoch, Vanessa Apea, Samuel Owiredu Hanson, Yaw Adu-Sarkodie

**Affiliations:** 1 School of Nursing, University of Rochester, Rochester, NY, United States of America; 2 Lawrence S. Bloomberg Faculty of Nursing, University of Toronto, Toronto, ON, Canada; 3 State University of New York at Binghamton, College of Community and Public Affairs, Department of Human Development, Binghamton, NY, United States of America; 4 Faculty of Humanities, University of Johannesburg, Johannesburg, South Africa; 5 Ghana Health Services, Ashanti Region Health Directorate, Kumasi, Ghana; 6 Cincinnati Children’s Hospital Medical Center, Division of Biostatistics and Epidemiology, Cincinnati, OH, United States of America; 7 University of South Florida, College of Nursing, Tampa, FL, United States of America; 8 Department of Genitourinary Medicine, Barts & the Royal London Hospital, London, United Kingdom; 9 Centre for Popular Education & Human Rights Ghana, Accra, Ghana; 10 Kwame Nkrumah University of Science & Technology, School of Medical Sciences, Kumasi, Ghana; The University of New South Wales, AUSTRALIA

## Abstract

Ghanaian men who have sex with men (MSM) have high rates of HIV infection. A first step in designing culturally relevant prevention interventions for MSM in Ghana is to understand the influence that peer social networks have on their attitudes and behaviors. We aimed to examine whether, in a sample of Ghanaian MSM, mean scores on psychosocial variables theorized to influence HIV/STI risk differed between peer social networks and to examine whether these variables were associated with condom use. We conducted a formative, cross-sectional survey with 22 peer social networks of MSM (n = 137) in Ghana. We assessed basic psychological-needs satisfaction, HIV/STI knowledge, sense of community, HIV and gender non-conformity stigmas, gender equitable norms, sexual behavior and condom use. Data were analyzed using analysis of variance, generalized estimating equations, and Wilcoxon two sample tests. All models were adjusted for age and income, ethnicity, education, housing and community of residence. Mean scores for all psychosocial variables differed significantly by social network. Men who reported experiencing more autonomy support by their healthcare providers had higher odds of condom use for anal (AOR = 3.29, p<0.01), oral (AOR = 5.06, p<0.01) and vaginal (AOR = 1.8, p<0.05) sex. Those with a stronger sense of community also had higher odds of condom use for anal sex (AOR = 1.26, p<0.001). Compared to networks with low prevalence of consistent condom users, networks with higher prevalence of consistent condom users had higher STD and HIV knowledge, had norms that were more supportive of gender equity, and experienced more autonomy support in their healthcare encounters. Healthcare providers and peer social networks can have an important influence on safer-sex behaviors in Ghanaian MSM. More research with Ghanaian MSM is needed that considers knowledge, attitudes, and norms of their social networks in the development and implementation of culturally relevant HIV/STI prevention intervention strategies.

## Introduction

Three decades since Human Immunodeficiency Virus (HIV) and Acquired Immune Deficiency Syndrome (AIDS) were first identified, men who have sex with men (MSM) in communities across the globe continue to be disproportionally affected by the HIV epidemic. In Ghana, the overall HIV prevalence decreased from 2.3% to 1.3% between 2010 and 2012 [1]. Comparatively, the HIV prevalence for Ghanaian MSM was 17.5%, indicating that MSM were nearly 15 times more likely to be infected with HIV than the general population [1]. Ghanaian MSM experience complex, multi-layered structural inequalities involving sexuality-related stigma and marginalization (e.g., criminalization of male-to-male sexual behavior), which hinders access to optimal health care and facilitates increased vulnerability to HIV risk [1–3]. Many West African governments, including Ghana, have only recently begun to recognize and take steps to address the prevention needs of MSM in their national HIV/AIDS strategies and control programs [4]. With the emerging emphasis on reducing HIV disparities for MSM in Ghana, more investigations are needed to contribute to a public health science evidence-base that will inform the development of culturally relevant HIV prevention.

The few existing studies on MSM in African countries share common findings indicating that core issues in HIV prevention for MSM involve low and inconsistent condom use during receptive and insertive anal intercourse, as well as low utilization of testing and treatment services for HIV and other sexually transmitted infections (STIs) [5–7]. Several systematic reviews focused on behavioral interventions specific to MSM report trends towards greater efficacy of interventions that leverage social interactions to promote behavior change [8–11]. For example, one systematic review of 102 studies showed that group and community-level interventions were associated with reductions in unprotected anal intercourse (UAI) and increased overall condom use among MSM [11]. In contrast, the evidence was inconsistent regarding the efficacy of individual-level interventions for reducing UAI among MSM [11].

HIV risk related attitudes, norms, and behaviors are produced within socio-cultural contexts where people are connected and interact [12]. The growing evidence of the impact of social networks on health risk and health promoting behaviors underscore the salience of investigating its relevance to sexual behavior and psychosocial infuencing factors for populations at high risk for HIV [13–17]. For example, in one study that examined the overlapping network of two Black MSM diagnosed with acute HIV infection, researchers found a 29% HIV prevalence in their shared sexual network (n = 398). Although the sexual networks included both men and women, the majority (98%) of HIV infections identified were among MSM [18]. In another study, local networks were used to examine differences in STI rates between Blacks and Whites in the United States [19]. The differences in STI prevalence were attributed to differences in the two groups’ social networking patterns. Social ties in a particular group also affect the flow of information, emotion, and influence in the group [20–23]. Furthermore, a given psychosocial phenomenon observed at the network-level is a product of the interactions between network members and cannot be understood as simply the sum of individual-level experiences [24].

Stigma is also an influencing factor on HIV/STI related sexual behaviors [25–28]. In Ghana, most studies on stigma have focused on investigating HIV stigma [29–32]. Several of these studies have consistently found that the experience of HIV stigma in Ghana involves social isolation [31,33], disapproval of HIV status disclosure [31–33], and financial instability related to HIV stigma in the workplace [30,32,33]. HIV stigma is not restricted to people living with HIV, but also has an impact on people closely associated with them—such as friends, relatives, and caregivers [31,32,34]. It is also common for HIV transmission to be associated with other behaviors that are socially marginalized, such as non-conformity to local gender norms [35–37]. While there is growing evidence on HIV stigma in Ghana, very little is known about its influence on HIV/STI risk related behaviors of MSM. For example, in a small qualitative study of MSM in Ghana, researchers found that the fear of healthcare providers stigmatizing them for being same gender practicing and suspecting them of having HIV was the primary barrier for MSM to seek HIV/STI prevention services [2]. Nonetheless, other studies on HIV stigma in Ghana have excluded analysis of MSM and the other interlocking forms of stigma that they likely experience (e.g., sexual stigma, gender non-conformity stigma).

Additionally, although most studies to date have focused on conceptualizations of stigma as a form of discrimination transmitted at the individual-level, recent studies have identified that stigma is a multi-level phenomenon that is also expressed in structural, forms [27,38–40]. In Ghana, structural stigma may be expressed in a number of ways. These include laws that criminalize same-sex behavior, institutionalized practices that undermine equal legal protection for MSM such as failure to arrest or prosecute perpetrators of anti-gay violence and penalizing people living with HIV/AIDS for non-disclosure of their HIV status even when condoms are used in sexual encounters. To date, only a few studies have been able to measure structural level stigmas; however, the available evidence suggests that structural-level stigmas interact with stigmas operating at the community and interpersonal levels (e.g., HIV stigma, gender non-conformity stigma) to exacerbate health disparities observed between marginalized groups and the general population [28,38,40–42].

This study had two overall objectives. The first objective was to examine whether psychosocial variables theorized to influence HIV/STI sexual risk behaviors differed between peer social networks in a sample of Ghanaian MSM. We hypothesized that mean psychosocial variable scores would differ between peer networks. The second objective was to examine whether, and to what degree, these psychosocial variables predicted condom use for anal, oral, and vaginal sex. For this objective, we hypothesized that higher psychosocial variable scores would be associated with increased condom use, with the exception of the stigma variables, which we expected to be associated with decreased condom use. We further hypothesized that peer social networks of high frequency condom users would have higher psychosocial variable scores than networks characterized by low frequency of condom use during sex.

## Theoretical Framework

**Figure 1:**
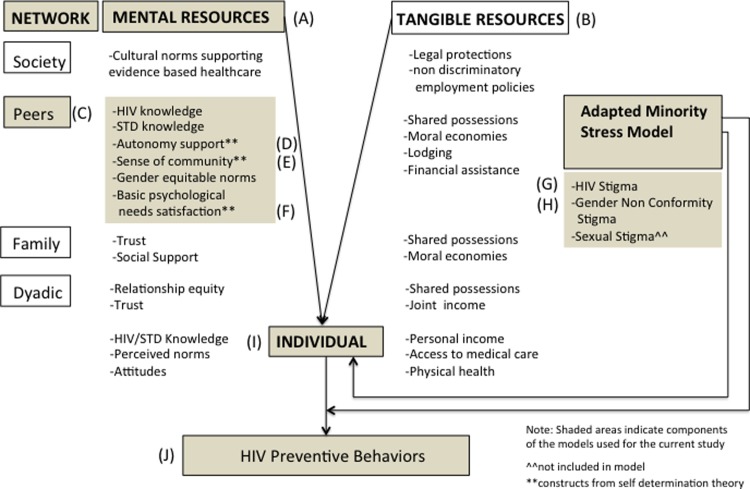
Integrated network-individual-resources (NIR), self-determination theory (SDT), and adapted minority stress framework. The (A) mental and (B) tangible HIV prevention resources that are in operation at the (C) peer network-level are theorized to influence (I) individuals and their enactment of (J) HIV preventive behaviors. The SDT concepts of (D) autonomy support, (E) sense of community and (F) basic psychological needs satisfaction are peer network-level mental resources within the NIR model. Within this integrated framework, (G) HIV stigma intersects with (H) gender non-conformity stigma to directly influence the (I) individual and also to moderate the influence of peer-network level mental resources on the individual’s (J) HIV preventive behavior.

The design of this study was informed by the network-individual-resource (NIR) model of HIV prevention with componets integrated from self-determination theory (SDT) and the adapted minority stress model (Fig. 1). Studying peer networks of MSM in Ghana requires culturally relevant theoretical and methodological approaches that can accomodate the conceptualization of individuals as interconnected units that comprise a group which has its own unified identities, attitudes, and norms. The NIR model is one recent innovation in HIV prevention theory development that takes into account the influence of close others, communities, and insitutional systems on an individual’s motivation for and enactment of HIV risk reduction behaviors (e.g., condom use, HIV/STI testing and treatment) [43]. This model also proposes that peer networks can affect health through social support, social behavioral regulation, access to resources and structuring exposure to HIV. In the NIR model there are both mental and tangible resources that must be met in order to optimize HIV prevention [43]. Mental resources refer to the psychsocial characteristics of people and networks that serve as assets for the avoidance of HIV/STI risk behaviors. Tangible resources are assets that structure conditions that facilitate healthy behaviors. In this study, we used NIR to guide the selection of variables reflecting constructs that serve as assets (e.g., HIV/STI knowledge, sense of community, gender equitable norms) to HIV prevention.

SDT is a social psychological theory of human motivation that contends that healthy behavior change is optimized in environments that support human’s basic psychological needs for autonomy, competence, and relatedness [44]. In this study, we integrated the SDT concepts of autonomy, competence, and relatedness into the NIR model by operationalizing them as mental resources of the peer networks that facilitate health behavior enactment (e.g., condom use) among its members. Specifically, we measured the degree to which participants experience of autonomy support from their peer networks and healthcare providers. Here, we wish to distinguish “independence” from the concept of “autonomy” as defined in SDT. Independence is the exercise of thought or action without external input. This is different from autonomy which involves the experience of one’s actions as fully willing and without external controls, while accomodating the input and influence of important others [45]. Therefore, as demonstrated in studies across various cultural contexts—including Brazil, Canada, China, India, Nigeria, Russia and Ukraine—SDT is congruent with collective-oriented cultures in that it does not presume “independence” in decision-making [46–50]. Decisions and/or behaviors that are derived from collective-centered thought can still be experienced as willful and volitional (not forced) with those involved assenting to act “together” or in the interest of the group. SDT accommodates the reality that, in Ghana, even while behaviors are expressed at the individual-level, antecedent psychoscial processes may at times be regulated at the group or community-level.

Finally, we integrated the adapted minority stress model into the NIR framework [36]. We operationalized HIV stigma and gender non-conformity stigma in the NIR model as threats which can undermine HIV prevention risk reduction behavior enactment. The original minority stress model posits that sexual stigma is associated with negative health outcomes for sexual minority people [51]. The adapted model, informed by intersectionality theory, extended the original model by including HIV stigma and gender non-conformity stigma with sexual stigma as interlocking experiences for MSM. In Ghana, like other countries in the West African region, men who exhbit mannerisms traditionally associated with women experience discrimination for not conforming to perceived cultural gender-based norms [2,5]. Moreover, people often associate gender non-conforming men with HIV infection, regardless of their actual sexual behavior or HIV status. This has been found in other studies with MSM and it important to assess the influence of these stigmas on safer sex behavior. [52,53].

## Methods

### Study Design

We conducted a cross-sectional survey of a non-probability sample of MSM peer networks. The study was conducted in the Accra, Koforidua/Manya Krobo, and Kumasi metropolitan areas of Ghana between March and June 2012. The Kwame Nkrumah University of Science & Technology (KNUST) Committee on Human Research, Publication and Ethics and the University of Toronto (UofT) HIV Research Ethics Board both approved the study. We obtained informed written consent from all participants. The purpose of the study and expectations for study participation were explained to all potential participants in the language in which they were most comfortable verbally communicating (e.g., English, Twi, Ga, Housa). We documented the informed consent by having participants sign their name directly onto an iPad-based electronic form via the touch screen. The digital signature was then uploaded via a secure 3G mobile network onto an encrypted database dedicated for the informed consent storage. We used digital signature and electronic storage of written informed consents in lieu of traditional paper/pencil methods. This was as an enhanced security procedure to protect the privacy of participants who enrolled in the study in a social context that was historically known to be hostile to same-sex behavior. The security-enhanced procedures for documenting informed consent were approved by UofT and KNUST.

### Eligibility and recruitment

Criteria for inclusion in the study were primarily at the network level—that is, we focused on network characteristics as the primary determinants of inclusion in the study. A peer network was eligible to be included in the study if (a) there was confidence among community-based organization (CBO) and KNUST-based outreach staff that the men were indicative of peer networks (i.e., friends), (b) there was confidence among the outreach staff that the men were MSM, and (c) at least four network members were willing to participate in the study. Potential participants who were interested in the study were asked to select up to 12 close friends from within their social network who were MSM and would be interested to participate in the study. Individual members of networks were excluded from participating in the study if they were younger than 18 years old or if they reported that they had not had anal or oral sexual intercourse with a man in the preceding six months.

We incorporated a community-based participatory approach in conducting the study [54]. For example, an interdisciplinary team of researchers, community research assistants (RAs), and community stakeholders with expertise and prevention experience with Ghanaian MSM worked collaboratively on all phases of the study, involving conceptualization, design, data collection, analyses, and interpretation. Local Ghanaian outreach staff from the CBO Centre for Popular Education & Human Rights Ghana and from the Kwame Nkrumah University of Science & Technology served as RAs. The RAs were multilingual and fluent in English and Twi, which are the two most widely spoken languages in the locations where the study was conducted. Some research assistants also had fluency in other languages that were spoken in local communities, such as Ewe, Fante, Ga, Hausa, and Krobo languages. The RAs were familiar with Ghanaian cultural and linguistic frameworks, which strengthened the cultural relevance in conducting this investigation.

We trained RAs on the research protocol as well as community site specific considerations. We also trained research them on the ethical conduct of research with human participants. This included training on informed consent, maintenance of participant privacy, data integrity, data security, research personnel and study participant safety, and emergency procedures. The RAs provided one-on-one information to each individual regarding the study procedures, including the risks of participation. The voluntary nature of the study was emphasized and participants were informed that they could withdraw from the study at any time for any reason. Participants were also informed that they would receive a one-time incentive of 25 New Ghana Cedis (GHS)—equivalent to approximately $14 US—for participating in the study. Furthermore, to reduce the risk of financial coercion, participants were informed that they would receive the incentive regardless of whether they completed the study.

### Data Collection

Each participant completed a survey consisting of a series of brief questionnaires that were administered using the iSurvey application on iPads. In total, eight questionnaires were administered. The survey included a variety of question and response formats from “true/false” questions designed to assess accuracy of knowledge and frequency questions (e.g., “never”, “a few times”, “most of the time”) that were designed to assess the frequency of exposures to stigma. We estimated that, on average, participants required 40-minutes to complete the survey. iSurvey does not include an audio-assisted component. Participants either self-administered the survey using the iPads or were assisted by RAs who either answered clarifying questions or read the questions and answer options to the participant, then allowed the participants to enter their answers and advance to the next screen without the RA observing the entry. Survey data were immediately uploaded via a mobile 3G network directly to a password encrypted database on a secure iSurvey server. Once the participant completed the survey, the data were no longer stored on the *i*Pad device and were not otherwise accessible to the RAs. This security feature minimized risk for privacy breaches, data loss, and database entry errors. This survey database was not linked to the informed consent database. All survey measures were in English. English is the official language of Ghana and the most accessible written language. Although non-English local languages are commonly spoken in casual everyday Ghanaian life, they are not as commonly written or read as English. We piloted the survey measures in Ghana to ensure that the constructs and their content were congruent with local Ghanaian understandings of gender, community, sex, and HIV/STI risk. During a pilot phase, we also assessed readability and comprehension by asking local volunteers to comment on the readability of the questionnaires and asked them to reflect back what was their understanding of each question. We used this information to slightly refine the wording of only a few items within the questionnaire. Nonetheless, the participants were provided the options of having survey items read to them or otherwise clarified in the language with which they were most comfortable verbally communicating.

### Measures

We collected demographic data to aid in understanding the composition of the peer networks. These nine items assessed important characteristics of age, ethnicity, community of residence, housing, income, HIV status, marital status, sexual attraction, and STI history. We used eight previously validated scales to assess psychosocial variables. Descriptions of the scales are summarized in Table 1.

**Table 1:** Summary Description of Scales Measuring Psychosocial Constructs.

					**Internal Consistency Reliability (α)**		
**Measure**	**Items**	**Scale Type**	**Values**	**Score Range**	**Current Study**	**Previous Studies**	**Sample Items**
Basic Needs Satisfaction Scale	9	Ordinal; 7-point Likert	Not at all true = 1	1–7	0.67	0.85–0.92	When I am with my friends, I feel free to be who I am? When I am with my friends I feel controlled and pressured to be certain ways
			Very true = 7				
Gender Non-Conformity Stigma Scale	13	Ratio	0 = Never	0–52	0.84	0.88	How often have you been made fun or called names because of your feminine mannerisms or behaviors? How often have you had to pretend that you were more masculine in order to be accepted?
			4 = Many times				
Healthcare Climate Questionnaire	15	Ordinal; 7-point Likert	1 = Strongly disagree	1–7	0.98	0.92	I feel that my healthcare provider accepts me. My healthcare providers try to understand how I see things before suggesting a new way to do things.
			7 = Strongly agree				
Brief HIV Knowledge Questionnaire	18	Ratio	Correct = 1	0–18	0.81	0.75–0.89	Can a person get HIV by sharing a glass of water with someone who has HIV?
			Incorrect = 0				
			I don’t know = 0				
Modified Felt HIV Stigma Scale	11	Ordinal	No one = 1	11–44	0.92	0.90	How many people think people with HIV are paying for their sins? How many people would not want an HIV-infected person cooking for them?
			1or 2 people = 2				
			A few people = 3				
			Most people = 4				
Sense of Community Scale	8	Ordinal; 5-point Likert	Strongly disagree = 1	8–40	0.87	0.92	I feel like a member of my network of friends. I can get what I need from my network of friends.
			Strongly agree = 5				
STD Knowledge Questionnaire	27	Ratio	Correct = 1	0–27	0.84	0.86	If a person has gonorrhea in the past, he or she is immune from getting it again
			Incorrect = 0				
			I don’t know = 0				
Gender Equitable Men Scale	27	Ordinal	Disagree = 1	27–81	0.88	0.79–0.81	It disgusts me when I see a man acting like a woman. Men need sex more than women do.
			Partially Agree = 2				
			Agree = 3				

The STD knowledge questionnaire (STDKQ) measures knowledge of sexually transmitted infections [55]. Participants responded “true”, “false”, or “I don’t know” to 27 statements regarding the transmission, prevention, and treatment of non-HIV STIs. Higher scores on the STDKQ indicate higher levels of STI knowledge. The brief HIV knowledge questionnaire (HIVKQ-18) is a measure of HIV transmission, prevention, and treatment. Originally a 45-item scale [56], we used the brief, 18-item version in order to reduce respondent burden [57]. Participants answered “true”, “false”, or “I don’t know” to statements regarding the transmission, prevention, and treatment of HIV.One point is given for each correct answer. Higher scores indicate higher levels of HIV knowledge.

We used the sense of community (SOCOM) scale to measure each members’ feeling of connectedness to their peer network [58]. The SOCOM scale assessed need fulfillment, group membership, influence, and emotional connection to a community or group. Higher scores indicate a greater sense of community with one’s social network. The basic needs satisfaction scale (BNSS) assessed SDT concepts of competence, autonomy, and relatedness that are theorized to need ongoing satisfaction for people to develop and function in healthy ways [59]. We used the BNSS to determine the degree to which members’ basic psychological needs were satisfied within their social networks. Higher scores indicate greater satisfaction of basic psychological needs within one’s peer network. We used the healthcare climate questionnaire (HCCQ) to assess the degree to which participants experience that their basic psychological needs for autonomy, competence, and relatedness were fulfilled in interactions with their health care providers. This construct allowed us to determine if network members had healthcare experiences that were optimized for health behavior change [60–62].

The modified felt normative HIV stigma (HIV stigma) scale was used to assess perceptions of the prevalence of HIV stigma within a community [63]. Participants were asked to report how many people in their communities shared the views reflected in statements regarding people living with HIV. Higher scores indicated higher degrees of felt normative HIV stigma. The gender non-conformity (GNC) stigma scale assessed the degree to which men felt devalued and/or treated negatively for displaying gender characteristics associated with women [36,64]. Participants indicated how often they experienced negative encounters related to being perceived as effeminate. We also used the gender equitable men scale (GEMS) to assess attitudes towards gender norms across various domains, including sexuality and sexual health [65]. The scale is composed of two subscales—one assesses attitudes towards inequitable gender norms (reverse scored) and the other assesses attitudes towards equitable gender norms. Higher sum total scores on both subscales indicate attitudes that are more supportive of gender equity.

We used six stand-alone items that recalled the number of total sexual encounters over the past three months versus the number of sexual encounters when condoms were used. We assessed the practice of condom use during anal, vaginal, and oral sex. For each sexual act type we defined condom use as whether or not (yes or no) the participant had ever used condoms over the past three months. These items have been used to assess risk in other sexual risk related studies [66,67].

### Analysis

We analyzed the survey data using SAS version 9.3. Measures of central tendency were used to summarize demographic data. We calculated means and standard deviations for the continuous and calculated frequencies for categorical variables.To address the first objective of the study, we used analysis of variance (ANOVA) to compare psychosocial variable scores between the peer networks.To investigate if pairs of networks were significantly different from each other, pairwise comparisons of knowledge and psychosocial scores between networks were performed using the Bonferroni method of multiple comparisons. To address our second objective to study the associations between condom use (for anal, oral, and vaginal sex) and psychosocial variables, we used generalized estimating equation (GEE) methods [68], which accounted for the clustering of individuals within social networks. We also adjusted for age, income, ethnicity, education, housing and community of residence in all models. We present the adjusted odds ratios (AORs) and the 95% confidence intervals. Finally, to see whether psychosocial variable scores differed by network condom use patterns we first classified the networks as either “high frequency” or “low frequency” condom users. We classified high frequency condom using networks as those in which all network members had greater than or equal to 50% condom use for all sexual act types. A peer network was classified as low frequency condom using if any member had < 50% rate of condom use for any sexual act type. We then used the Wilcoxon two-sample test to compare the mean psychosocial variables scores between high and low frequency condom using networks.

## Results

A total of 22 MSM peer networks enrolled in the study (n = 137). The networks ranged in size from four to eight MSM, with an average network size of 6.23. Table 2 presents a demographic summary of the sample. The men ranged in age from 18 to 55, with a median age of 24.65 (*SD 5.4*). The group was predominantly low income. The mean monthly income of 13,115 GHS ($4,035 US dollars) was skewed high due to a few very high income-earning participant in the sample. The median monthly income was 70 GHS ($21 US dollars). There were nine ethnicities represented among the MSM in the study. The Asante were the most prevalent ethnic group in the sample, followed by the Ga and Krobos. These three ethnicities accounted for 77.4% of the study sample. Most of the men in study were only sexually attracted to other men, although a substantial (42%) proportion of the sample reported that they were also sexually attracted to women.

**Table 2:** Descriptive Statistics for Demographic Variables (N = 137).

**Demographics**	**%**
**Age (years)**	
18–24	59.1
25–34	37.3
35–44	2.8
45 and over	0.7
**Ethnicity**	
Asante	40.9
Ga	19
Krobo	17.5
Multi-Ethnic	5.1
Ewe	4.4
Hausa	2.9
Akuapem	2.2
Akyem	2.2
Fante	1.5
**Community of Residence**	
Accra	38.7
Kumasi	37.2
Manya Krobo	25.2
**Housing**	
Living with parents	47.4
Renting	28.5
Boarding House	17.5
Living in own home	5.8
Squatting	0.7
**HIV Status**	
Seronegative	75
Serpositive	0.7
HIV status unknown	23
Decline to answer	2
**Marital Status**	
Married	3.6
Not Married	96.4
**Sexual Attraction**	
Men Only	56.9
Women Only	0.7
Both Men and Women	42.3
**Lifetime STD History**	
No	72
Yes	28

The mean scores on psychosocial variables for the overall sample are presented in Table 3. Overall, the men had low knowledge of STI transmission, prevention and treatment and a moderate level of knowledge regarding transmission, prevention, and treatment of HIV. There was also a low mean score for GNC stigma indicating that in the overall sample the experience of stigma related to exhibition of characteristics or mannerisms associated with women and femininity was low. The participants felt a moderately high level of HIV stigma in the communities where they lived. The sense of community and satisfaction of basic psychological needs within the social networks was generally high in the overall sample. The mean HCCQ score indicated that the men felt a relatively moderate degree of basic psychological needs satisfaction, and consequently autonomy support from their healthcare providers. The GEMS mean score also indicated that gender norms in the overall sample were equity supportive. All scales demonstrated good to excellent internal consistencies, except for the basic need satisfaction scale, which demonstrated acceptable internal consistency (α = 0.67).

**Table 3:** Summary of psychosocial variable scores for overall sample.

**Measure (N = 137)**	**Mean**	**SD**
STD Knowledge Questionnaire (STDKQ)	12.2	5.7
HIV Knowledge Questionnaire (HIVKQ)	12.0	3.7
Sense of Community Scale (SOCOM)	36.1	4.4
Healthcare Climate Questionnaire (HCCQ)	5.1	1.5
Gender Non Conformity stigma scale (GNC Stigma)	21.1	6.7
Felt Normative HIV stigma scale (HIV Stigma)	24.1	7.3
Basic need satisfaction scale (BNSS)	5.6	0.7
Gender Equitable Men’s Scale	51.7	6.6

The results of the ANOVA comparing all 22 groups are summarized in Table 4. There were statistically significant differences between peer networks on all and psychosocial variables, with p<0.0001 in all cases except GNC stigma (p = 0.044). The means and standard deviations for psychosocial mean scores across the 22 networks are presented in Tables 5 and 6. The social networks located in Accra were not significantly different from each other. The social networks in Kumasi were all statistically significantly different from each other, as were the networks in Manya Krobo. Social networks did not differ on reports of condom use in the past three months for oral, anal, or vaginal sex.

**Table 4:** Results of ANOVA Comparing Peer Network Mean Scores on Psychosocial Variables.

**Variable**	**Sums of Squares**	**Mean Square**	**F (21, 136)**	**p-value**
STD Knowledge				
Between groups	2553.58	121.60	7.58	<.0001
Within groups	1844.10	16.04		
HIV Knowledge				
Between groups	732.41	34.88	3.52	<.0001
Within groups	1138.60	9.90		
Sense of Community				
Between groups	1450.74	69.08	6.53	<.0001
Within groups	1216.34	10.58		
Healthcare Climate				
Between groups	255.28	12.16	19.68	<.0001
Within groups	71.05	0.62		
Gender Non Conformity Stigma				
Between groups	1434.05	68.29	1.70	0.0405
Within groups	4622.31	40.19		
Felt Normative HIV stigma				
Between groups	4505.87	214.57	9.18	<.0001
Within groups	2689.02	23.38		
Basic Need Satisfaction				
Between groups	27.27	1.30	3.49	<.0001
Within groups	42.81	0.37		
Gender Equitable Norms				
Between groups	3579.09	170.43	8.59	<.0001
Within groups	2280.37	19.83		

**Table 5:** Means and standard deviations on psychosocial variables for peer networks 1–11.

**Measure**	**Peer Social Network (network size)**										
	**1 (n = 6)**	**2 (n = 8)**	**3 (n = 5)**	**4 (n = 6)**	**5 (n = 6)**	**6 (n = 6)**	**7 (n = 8)**	**8 (n = 6)**	**9 (n = 8)**	**10 (n = 6)**	**11 (n = 6)**
	**M (SD)**	**M (SD)**	**M (SD)**	**M (SD)**	**M (SD)**	**M (SD)**	**M (SD)**	**M (SD)**	**M (SD)**	**M (SD)**	**M (SD)**
STDKQ	14.83 (1.5)	16 (1.3)	16.2 (1.1)	13.2 (1.7)	17.2 (3.7)	19.8 (1.9)	17.9 (3.3)	18.7 (1.6)	7.8 (4.3)	12.2 (3.9)	13.3 (5.7)
HIVKQ	12 (0.9)	13.6 (1.6)	13.8 (0.8)	12.7 (1.4)	14.2 (1.2)	15.2 (0.4)	14.5 (1.1)	15 (0.0)	10.8 (3.6)	14.0 (2.4)	12.5 (2.9)
SOCOM	35.7 (2.7)	36.4 (2.8)	36.6 (3.0)	37.7 (2.2)	37.0 (0.9)	38.8 (1.1)	39.1 (1.7)	39.2 (0.8)	32.9 (3.8)	30.2 (4.4)	35.0 (2.4)
HCCQ	3.6 (0.7)	3.9 (0.3)	4.1 (0.1)	2.1 (0.9)	3.8 (0.1)	3.8 (0.1)	3.6 (1.0)	3.9 (0.1)	4.6 (1.3)	5.6 (1.0)	5.3 (0.8)
GNC Stigma	21.2 (7.7)	23.0 (6.2)	21.2 (5.5)	22.3 (7.0)	19.7 (6.6)	20.3 (6.0)	20.6 (5.4)	19.7 (5.7)	28.8 (6.9)	21.2 (3.5)	23.0 (3.3)
HIV Stigma	17.3 (1.5)	19.4 (3.0)	19.8 (2.5)	30.8 (1.0)	21.0 (5.7)	18.8 (3.3)	21.3 (5.1)	21.0 (3.4)	28.1 (6.0)	26.2 (5.7)	30.5 (2.4)
BNSS	5.4 (0.3)	5.3 (0.4)	5.6 (0.1)	5.4 (0.2)	5.5 (0.3)	5.7 (0.2)	5.7 (0.2)	5.9 (0.1)	5.6 (0.9)	5.4 (0.9)	6.0 (0.3)
GEMS	45.0 (1.5)	45.3 (1.8)	45.2 (1.6)	56.0 (2.0)	48.5 (3.9)	44.8 (2.1)	46.1 (3.7)	46.8 (2.3)	58.9 (7.9)	57.2 (6.8)	55.5 (7.2)

Note: Peer networks 1–8 were sampled from Kumasi and peer networks 9–11 were sampled from Accra.

**Table 6:** Means and standard deviations on psychosocial variables for peer networks 12–22.

**Measure**	**Peer Social Network (network size)**										
	**12 (n = 7)**	**13 (n = 6)**	**14 (n = 6)**	**15 (n = 6)**	**16 (n = 8)**	**17 (n = 4)**	**18 (n = 6)**	**19 (n = 6)**	**20 (n = 7)**	**21 (n = 6)**	**22 (n = 4)**
	**M (SD)**	**M (SD)**	**M (SD)**	**M (SD)**	**M (SD)**	**M (SD)**	**M (SD)**	**M (SD)**	**M (SD)**	**M (SD)**	**M (SD)**
STDKQ	11.4 (4.5)	10.2 (5.5)	14.7 (5.70)	8.2 (3.4)	9.8 (2.8)	10.0 (5.4)	8.0 (4.6)	9.2 (6.2)	3.6 (5.8)	6.3 (3.3)	10.0 (4.7)
HIVKQ	10.9 (4.2)	12.2 (2.3)	14.3 (3.2)	11.2 (2.3)	10.0 (2.9)	9.0 (6.2)	10.8 (4.5)	10.5 (6.3)	5.4 (5.6)	10.0 (3.0)	12.0 (1.4)
SOCOM	30.7 (4.9)	39.0 (1.5)	31.2 (8.0)	40.0 (0.0)	38.0 (3.9)	33.5 (2.6)	30.3 (5.3)	35.5 (2.9)	40.0 (0.0)	39.5 (1.2)	38.8 (2.5)
HCCQ	5.0 (1.5)	6.1 (0.9)	6.0 (1.8)	5.7 (0.3)	5.8 (0.2)	7.0 (0.1)	7.0 (0.1)	6.7 (0.4)	6.9 (0.1)	6.8 (0.3)	6.7 (0.1)
GNC Stigma	19.6 (5.6)	23.5 (14)	28.7 (14)	21.8 (2.0)	21.1 (4.5)	18.3 (1.0)	17.2 (1.6)	15.8 (8.2)	16.4 (1.6)	16.0 (2.7)	18.8 (3.8)
HIV Stigma	31.4 (4.2)	32.7 (0.8)	27.8 (8.8)	33.0 (0.0)	31.4 (3.5)	14.3 (7.1)	19.2 (3.5)	19.2 (3.5)	19.4 (6.2)	24.0 (3.4)	20.8 (9.3)
BNSS	5.4 (0.6)	5.8 (0.3)	5.4 (1.0)	6.4 (0.5)	6.4 (0.5)	4.9 (0.5)	4.2 (1.7)	5.4 (0.7)	5.8 (0.1)	5.7 (0.5)	5.8 (0.5)
GEMS	56.9 (7.7)	55.1 (3.5)	51.8 (5.8)	50.7 (1.8)	48.3 (1.9)	51.3 (4.7)	49.3 (6.0)	51.3 (2.0)	57.4 (3.8)	60.7(0.8)	57.5 (5.3)

Note: Peer networks 12–16 were sampled from Accra and peer networks 17–22 were sampled from Manya Krobo.

The GEE models are summarized in Table 7. The GEE models indicated that the STDKQ, HCCQ, SOCOM and HIV stigma variables were associated with an increased likelihood of condom use. High STI knowledge was associated with increased likelihood of condom use for oral sex. The HCCQ was associated with an increased likelihood of condom use for oral sex. HIV Stigma was also associated with increased likelihood of condom use for oral sex. Experiencing greater autonomy support by one’s healthcare provider was associated with a more than three-fold odds of condom use for anal sex. Strong sense of community was also associated with improved likelihood of condom use for anal sex. Higher gender equitable norms were associated with increased odds of condom use for anal and oral sex. None of the remaining psychosocial variables were associated with condom use for anal sex. High scores on STI knowledge and health care climate were associated with increased likelihood of using condom during vaginal sex. Finally, the network level analysis (Table 8) revealed that high frequency condom using peer networks had significantly higher STD knowledge, HIV knowledge, and gender equitable norms than low frequency condom using networks. Additionally, low frequency condom using peer networks experienced less autonomy support in their healthcare encounters compared to high frequency condom using networks.

**Table 7:** Generalized estimating equation assessing associations between social and psychological variables and condom use.

	**Condom Use**		
			
	**^a^Odds Ratio (95% CI)**		
			
**Measure *(N = 137)***	**Oral Sex**	**Anal Sex**	**Vaginal Sex**
STD KQ	1.55 (1.17, 2.05)^c^	1.20 (0.95, 1.50)	1.29 (1.08, 1.55)^c^
HIV KQ	1.34 (0.98, 1.83)	1.09 (0.86, 1.39)	1.37 (0.94,1.98)
SCS	1.13 (0.93, 1.37)	1.26 (1.05, 1.52)^d^	1.06 (0.90, 1.27)
HCCQ	5.06 (1.75, 14.63)^c^	3.29 (1.42, 7.66)^c^	1.8 (1.06, 3.06)^d^
GNS	1.01 (0.94, 1.09)	1.02 (0.97, 1.08)	0.95 (0.84, 1.08)
HIV Stigma	1.26 (1.11, 1.43)^b^	1.11 (0.99, 1.24)	1.11 (0.99, 1.24)
Basic Needs Satisfaction	2.70 (0.64, 11.47)	1.25 (0.46, 3.39)	1.25 (0.46, 3.39)
Gender Equitable Norms	1.27 (1.06, 1.51) ^c^	1.15 (1.02, 1.30) ^d^	1.15 (0.98, 1.36)

^a^ Adjusted for age, income, ethnicity, education, housing and community of residence.

^b^ p<0.001.

^c^ p<0.01.

^d^ p<0.05.

**Table 8:** Comparison of Low and High Frequency Condom Using Peer Social Networks on Psychosocial Variable Scores.

**Measure (Mean, SD)**	**Network Condom Use Frequency Classification**		
	**Low (n = 9)**	**High (n = 13)**	**p^a^**
STD Knowledge Questionnaire	10.1 (2.3)	15.3 (4.8)	0.0041
HIV Knowledge Questionnaire	11.4 (1.6)	12.9 (3.0)	0.0252
Sense of Community Scale	35.0 (3.8)	37.8 (1.5)	0.0885
Healthcare Climate Questionnaire	4.0 (1.2)	6.0 (0.8)	0.0017
Gender Non Conformity Stigma Scale	20.5 (1.9)	21.3 (3.9)	0.9733
Felt Normative HIV Stigma Scale	21.0 (3.9)	25.8 (6.4)	0.1089
Basic need satisfaction scale	5.6 (0.2)	5.6 (0.6)	0.9202
Gender Equitable Men’s Scale	48.4 (4.9)	54.2 (3.9)	0.0092

^a^ p-value based on two-sided Wilcoxon two-sample test.

## Discussion

HIV prevention research focused on Ghanaian MSM has been a significantly understudied area of scientific inquiry, contributing to a limited understanding of their HIV prevention needs, including culturally relevant considerations that influence condom use. Much of the research in this area indicates that a number of HIV prevention programs for African, including Ghanaian, MSM are largely informed by imported evidence based on studies from other geographical contexts reflective of Western modalities [8–10,69] or African-descendant MSM in communities outside of the continent [11,27]. This study’s objectives were to investigate whether peer social networks differed in mean scores on HIV/STI related psychosocial variables and to examine whether these variables were associated with condom use in a sample of Ghanaian MSM.

Our results provide formative evidence that can be used to inform HIV prevention strategies with MSM in Ghana. Moreover, this study contributes to the growing literature on peer network influences on sex and sex-related constructs in MSM [12,18,70–72]. To date, this is the first known study in Ghana to focus on peer social networks of MSM and to demonstrate that psychosocial influencing factors for condom use are clustered within different peer networks. Like previous studies, our focus on social networks of MSM did not limit the network configurations to sexual partners [12,73]. By focusing on MSM peer networks (i.e., friends), we may have been able to capture a broader range of non-sexual social influence on sexual behavior of MSM in our sample. In addition, our study focused on understanding predictors of safer sex behavior versus predictors of HIV risk, which is a common focus of research on MSM [7,74]. Our focus on characteristics that support condom use is a purposeful application of a strength-based and integrative anti-racism approach that resists the hyper-emphasis on risk behaviors of African and African-descendant MSM in HIV prevention research and advances more balanced characterizations of African men’s sexualities and sexual health related outcomes [75–77].

We found that STI knowledge, HIV knowledge, and the other psychosocial variables differed by social network. This provides important evidence for how networks can be better utilized as the focus of sexual health promotion and risk reduction interventions. One potential explanation for these results is that MSM with similar knowledge levels, attitudes, and experiences cluster together in similar groups such that network-level phenomenon is a function of individuals [78]. Another explanation is that individuals that compose the network generate—through group acculturation and socialization processes—attitudes, negotiated knowledge, and shared (direct or indirect) social experiences that form a network mentality with its own associated rationality [20,43]. We believe that the latter explanation is the most plausible, based on corroborating evidence from the literature, and is precisely why network-focused interventions hold promise for optimizing HIV prevention for MSM in Ghana [5,12,16,71]. That is, if influencing factors for condom use are found to also be operating at the network-level, then strategies that seek to target these constructs and motivate change at network-level are warranted. Given the complexity of HIV prevention for MSM within a sociocultural context where their sexualities are marginalized and their sexual behaviors are stigmatized, social networks offer an additional opportunity through which to pursue the development of innovative strategies that support reductions in new infections.

HIV knowledge was not associated with condom use for any sexual behaviors. MSM with higher STI knowledge levels had increased odds of condom use for oral or vaginal sex. This finding may suggest that knowledge of the other non-HIV sexually transmissible infections, such as gonorrhea, may contribute to increased condom use for oral and vaginal sexual activities [55,79]. It could also suggest a disproportionate emphasis in STI education on heterosexual sexual activities, which may be perceived to only involve either oral or vaginal sex. Moreover, it highlights the need for integrated HIV and STI health education since HIV knowledge alone may not be sufficient to motivate sexual risk-reduction for MSM. We note that, in comparison to oral and vaginal sex, increased STI knowledge was not associated with increased condom use for anal sex. This finding may reflect a lack of sufficient attention to anal sex in STI education in Ghana. It is plausible that anal sex is stigmatized and excluded from health education programs precisely because of its association with same-gender sexual behavior, representing a structural discriminatory barrier to prevention [80]. Furthermore, it is possible that comprehensive information regarding STI risk involved with unprotected anal intercourse (UAI) are excluded from the health education provided by health care providers, who are important sources of credible health information. The withholding of such vital health information is inconsistent with SDT as doing so undermines autonomy and competence, since it does not allow MSM to make their own decisions based on the best available scientific evidence [81–83]. Evidence-based, culturally relevant HIV/STI prevention programs that have strong educational components, with specific attention to risk associated with UAI, can help support the safer sex in Ghanaian MSM [79,84,85]. These programs can be implemented through CBOs that focus on providing services to MSM clients [2]. Additionally, efforts must be made to intervene with healthcare providers to increase their dissemination of adequate health information to their MSM patients so that these men are equipped with the pre-requisite knowledge necessary to make informed decisions regarding their sexual behaviors [61,86,87].

Men who reported that their basic psychological needs for autonomy, competence, and relatedness were satisfied in their encounters with their health were more likely to use condoms for oral, anal, and vaginal sex. Men who felt a strong sense of community within their peer networks were also more likely to use condoms for anal sex. These findings are consistent with the NIR and SDT theoretical models. Based on our knowledge, these results are among the first empirical evidence establishing the impact of peer network dynamics and the salience of basic psychological-needs satisfaction in healthcare provider approaches on safer sexual behaviors of MSM in Ghana. The SDT proposition that the clients’ experiences of their providers as supportive of their basic psychological needs are associated with healthy behavior change and maintenance is grounded in a well-established literature across a number of health domains [81,86,88,89]. Nonetheless, there are only a few known studies of its application in the HIV/STI prevention domain [90,91]. Many HIV prevention studies for MSM focus on targeting modification in the men’s behavior. The results of this study suggest the need for structural interventions that transform healthcare environments from controlling ones that dictate expected health behaviors to ones that support MSM to exercise informed choices in their health behavior decisions [92]. Future studies should test interventions designed to enhance the autonomy supportiveness of healthcare provider approaches to working with MSM in Ghana. As we have demonstrated in this study, SDT can also be used to operationalize concepts in some of the new and emerging HIV prevention models such as the NIR model. Furthermore, its articulation of the three basic psychological needs provides a theoretically grounded explanation for the links found in previous studies between social connectedness, social support, and decreased sexual risk behavior in MSM [93,94]. From a SDT perspective, social support and connectedness would be associated with increased safer sex behavior because it satisfies the basic psychological need for relatedness, which is one’s feeling of being valued and respected by close others whom one respects and values—to the extent that those others endorse safer-sex behaviors [44,87].

At the structural level, SDT can be used to guide the development of interventions targeting policies that purport to be aimed at reducing the spread of HIV, but nonetheless perpetuate inequities. The results from a number of studies demonstrate that coercive policies (e.g., surveillance) and practices (e.g., penalties) that exert control and exact punishment are less effective, over the long-term, at maintaining their intended behavioral goals [95]. For example, HIV prevention policies that are experienced as oppressive, such as those that threaten punishment for non-disclosure of HIV status, may have the short-term impact of compelling disclosure. However, there is evidence that, over time, such policies generate distress, defiance, and opposition, among those populations who are subject to it, which in turns require increased punishments to maintain compliance [96–99]. Successful structural interventions for HIV prevention have been ones that have increased access to resources and prevention options for high-risk groups [100–104]. Instead of motivating by implementing policies intended to scare, autonomy supportive policies would provide meaningful information about why the intended behavior is sought, clear rationale for how it helps achieve local community goals (or by how not enacting the behaviors undermines community goals), and emphasis on quality options from which to choose [61,95,105]. More research is needed to evaluate the effects of SDT based interventions for improving primary and secondary prevention outcomes by targeting policies that perpetuate structural stigma on MSM.

While there are a limited number of studies has focused on MSM in Ghana, our findings are consistent with previous research studies of Black MSM in the US [72,106–108]. For example, in one study of Black MSM (N = 197) in the Boston metropolitan area, researchers found that socially isolated men were more likely (AOR = 4.23, *p*<0.05) to have engaged in UAI with an HIV-positive male partner in the past year [72]. Findings also demonstrated that men who reported peer social norms supporting condom use were also less likely (AOR = 0.60; *p* = 0.05) to report having had UAI in the past year. Another study of Black MSM (N = 210) from three US cities (Cleveland, Miami, Milwaukee) found that those who reported peer norms that were weakly supportive of condom use had greater frequency of UAI [106]. In the US, a number of peer-based interventions have been developed and tested for MSM and demonstrated efficacy in reducing HIV infections by increasing condom use and reducing number of sexual partners, both methods which serve to reduce the number of potential exposures to HIV [9,84,109]. More research is needed to understand whether promoting a sense of community within MSM social networks and working with healthcare providers to facilitate autonomy-supportive healthcare environments for MSM can strengthen the efficacy that both established and emerging interventions have on behavioral and clinical outcomes. For example, the Many Men, Many Voices (3MV) intervention which has been disseminated and used with African Diaspora MSM in the US and Canada does not presuppose that the men who participate are close friends or have any knowledge of one another upon entry into the intervention sessions [85]. Targeting social networks for the 3MV intervention may be an important innovation to the existing intervention model that may enhance its efficacy on behavioral outcomes. Moreover, research is needed to develop homegrown interventions that are grounded in Ghanaian sociocultural experiences and integrated within Ghanaian health care systems, thus building on the utilization of peer networks and health care providers as key mechanisms to leverage HIV/STI risk reduction motivation and maintenance.

Last, cultural worldviews and values as a core component of conceptual frameworks are integral to the development and implementation of primary and secondary HIV prevention intervention strategies [110]. For many Western cultural frameworks, the concept of “self” is conceptualized as independent and unique of others in their social milieu. In cultures where self-construal is collective-centered, such as those in Ghana, self may be experienced as interdependent to the group(s) to which one belongs [111–113]. For example, the role of friends, families, extended families, and communities in African cultural frameworks are salient in connection to understanding sexuality, health behavior, cultural beliefs, and health-related attitudes [70]. Therefore, conceptualizations of sex-related attitudes and norms based primarily on individual-level phenomena, as situated in Eurocentric or Western cultural frameworks, limit researchers’ abilities to incorporate collective-centeredness as a sociocultural strength-based domain that can be utilized to enhance HIV prevention efforts with MSM in Ghanaian communities. More research that utilizes frameworks such as the NIR model are needed in order to build the evidence-base regarding the multi-level factors influence HIV risk reduction behaviors in Ghanaian MSM.

There are key limitations to this research study. First, we used a cross-sectional study design and, as such, the relationships between the psychosocial and condom use variables must not be viewed as causal. The data are self-report and subject to social desirability bias. We used the self-administered iSurvey to help minimize the degree to which this bias may have been introduced into the dataset. We also recruited a non-probability sample of MSM and thus the participants in our study may not reflect the larger population of MSM in Ghana. Nonetheless, we present important preliminary evidence that experience of autonomy support in interactions with healthcare providers and a strong sense of community within one’s social network were associated with greater of condom use by MSM in Ghana.

## Conclusion

Our results contribute to an evidence-base foundation upon which future studies of Ghanaian MSM can be developed. Even as researchers call for combination interventions to address the HIV prevention [6] among MSM in Africa, it will be important to consider innovative implementation strategies that will maximize uptake and leverage cultural assets [114]. The literature on basic psychological-needs satisfaction is well established but is new in its application to HIV/STI prevention related behaviors. Programs that enhance sense of community within peer networks and that target healthcare providers’ approaches to working with MSM may find that those networks themselves are prime considerations for interventions designed to yield HIV risk reduction behavioral and clinical outcomes.
